# Mild thrombocytopenia indicating maternal organ damage in pre‐eclampsia: a cross‐sectional study

**DOI:** 10.1186/s12884-021-03564-4

**Published:** 2021-01-28

**Authors:** Michinori Mayama, Mamoru Morikawa, Takashi Yamada, Takeshi Umazume, Kiwamu Noshiro, Kinuko Nakagawa, Yoshihiro Saito, Kentaro Chiba, Satoshi Kawaguchi, Hidemichi Watari

**Affiliations:** 1 Department of Obstetrics and Gynecology, Hokkaido University Graduate School of Medicine, Kita-ku N15 W7, 060-8638 Sapporo, Hokkaido Japan; 2 Department of Obstetrics and Gynecology, Japan Community Health Care Organization Hokkaido Hospital, Toyohira-ku, Nakanoshima 1-8, Sapporo, Hokkaido Japan

**Keywords:** Cut-off value, Maternal organ damage, Pre-eclampsia, Preterm delivery, Thrombocytopenia

## Abstract

**Background:**

Currently, there is a disagreement between guidelines regarding platelet count cut-off values as a sign of maternal organ damage in pre-eclampsia; the American College of Obstetricians and Gynecologists guidelines state a cut-off value of < 100 × 10^9^/L; however, the International Society for the Study of Hypertension in Pregnancy guidelines specify a cut-off of < 150 × 10^9^/L. We evaluated the effect of mild thrombocytopenia: platelet count < 150 × 10^9^/L and ≥ 100 × 10^9^/L on clinical features of pre-eclampsia to examine whether mild thrombocytopenia reflects maternal organ damage in pre-eclampsia.

**Methods:**

A total of 264 women were enrolled in this study. Participants were divided into three groups based on platelet count levels at delivery: normal, ≥ 150 × 10^9^/L; mild thrombocytopenia, < 150 × 10^9^/L and ≥ 100 × 10^9^/L; and severe thrombocytopenia, < 100 × 10^9^/L. Risk of severe hypertension, utero-placental dysfunction, maternal organ damage, preterm delivery, and neonatal intensive care unit admission were analyzed based on platelet count levels. Estimated relative risk was calculated with a Poisson regression analysis with a robust error.

**Results:**

Platelet counts indicated normal levels in 189 patients, mild thrombocytopenia in 51 patients, and severe thrombocytopenia in 24 patients. The estimated relative risks of severe thrombocytopenia were 4.46 [95 % confidence interval, 2.59–7.68] for maternal organ damage except for thrombocytopenia, 1.61 [1.06–2.45] for preterm delivery < 34 gestational weeks, and 1.35 [1.06–1.73] for neonatal intensive care unit admission. On the other hand, the estimated relative risks of mild thrombocytopenia were 0.97 [0.41–2.26] for maternal organ damage except for thrombocytopenia, 0.91 [0.62–1.35] for preterm delivery < 34 gestational weeks, and 0.97 [0.76–1.24] for neonatal intensive care unit admission.

**Conclusions:**

Mild thrombocytopenia was not associated with severe features of pre-eclampsia and would not be suitable as a sign of maternal organ damage.

## Background

Hypertensive disorders of pregnancy (HDP) can cause hypertension and maternal organ damage due to endothelial dysfunction [1, 2]. Among patients with HDP, women who develop maternal organ damage in addition to hypertension are diagnosed with pre-eclampsia, even if they do not develop proteinuria. Coagulation disorders are considered a sign of maternal organ damage in preeclamptic patients [1, 2]. Thrombocytopenia is the most frequently detected coagulation disorder in pre-eclampsia and likely occurs as a result of the injured endothelium activating platelets, leading to elevated consumption of platelets [3, 4]. Pre-eclampsia is the most common cause of thrombocytopenia with evidence of thrombotic microangiopathy in the second and third trimester of pregnancy [5].

Severe thrombocytopenia: platelet count (PC) < 100 × 10^9^/L is a sign of severe pre-eclampsia, and termination of pregnancy should be considered [1]. However, the clinical significance of mild thrombocytopenia (≥ 100 × 10^9^/L and < 150 × 10^9^/L) in pre-eclampsia remains controversial. The International Society for the Study of Hypertension in Pregnancy (ISSHP) guidelines state PC levels < 150 × 10^9^/L as a sign of maternal organ damage, whereas the American College of Obstetricians and Gynecologists guidelines state a cut-off value of < 100 × 10^9^/L [1, 2]. Although termination of pregnancy must be considered in the context of gestational age and other clinical and laboratory results in combination with PC levels, the classification of HDP is affected by whether mild thrombocytopenia is included as a sign of maternal organ damage. Therefore, the discrepancy in PC cut-off values between the two major guidelines makes it difficult to compare studies based on these different guidelines. Actually, a sign of maternal organ damage, including thrombocytopenia (< 150 × 10^9^/L), was included in the criteria of classification of HDP in May 2018 in Japan and it increased the number of women who were diagnosed with pre-eclampsia [6].　To assess whether mild thrombocytopenia reflects maternal organ damage in patients with pre-eclampsia, we examined the impact of mild thrombocytopenia on the severity of pre-eclampsia and perinatal outcomes.

## Methods

### Study Design and Study Population

This retrospective cross-sectional study was conducted at Hokkaido University Hospital and Japan Community Health Care Organization Hokkaido Hospital, and medical records of women whose delivery happened between April 2010 and May 2019 in the two hospitals were reviewed. Women diagnosed with HDP were included, and the following exclusion criteria were applied in this study: <18 years old at delivery, transferred to other hospitals before delivery, thrombocytopenia not related to HDP, severe congenital anomaly in the baby, twin pregnancy, and insufficient data. Among the patients with HDP, women diagnosed with gestational hypertension and chronic hypertension were also excluded.

### Data Collection

According to ISSHP and Japan Society for the Study of Hypertension in Pregnancy guidelines, pre-eclampsia was defined by the new onset of hypertension (systolic blood pressure ≥ 140 mmHg or diastolic blood pressure ≥ 90 mmHg) accompanied by proteinuria (protein/creatinine ratio ≥ 0.3) and/or a sign of maternal organ damage and utero-placental dysfunction, as listed in Table 1. The women with pre-eclampsia were categorized into three groups according to PC levels at delivery: normal, PC ≥ 150 × 10^9^/L; mild thrombocytopenia, ≥ 100 × 10^9^/L and < 150 × 10^9^/L; and severe thrombocytopenia, < 100 × 10^9^/L. We analyzed the prevalence of severe hypertension (systolic blood pressure ≥ 160 mmHg or diastolic blood pressure ≥ 110 mmHg), maternal organ damage, utero-placental dysfunction, and gestational age at the onset of pre-eclampsia, as well as the rate of neonatal intensive care unit (NICU) admission and preterm delivery < 34 gestational weeks (GW).

**Table 1:** The criteria of maternal organ damage and utero-placental dysfunction

λ Liver involvement without underlying diseaseAST or ALT > 40 IU/L with or without right upper quadrant or epigastric abdominal pain
λ Acute kidney injury Serum creatine level ≥ 90 umol/L; 1.0 mg/dL
λ Neurological complications Eclampsia, altered mental status, blindness, stroke, clonus, severe headache, or persistent visual scotoma
λ Blood coagulation disorders Thrombocytopenia: platelet count < 150 × 10^9^/L, disseminated intravascular coagulation, or hemolysis
λ Utero-placental dysfunction Fetal growth restriction^a^, abnormal umbilical artery doppler waveform^b^, or stillbirth^c^

^a^: Estimated fetal weight < − 1.5 standard deviation without chromosomal abnormality and multiple congenital anomaly syndrome

^b^: Absent or reversal of end diastolic flow, or extremely high pulsatility index or resistance index

^c^: The cases of stillbirth with chromosomal abnormality or multiple congenital anomaly syndrome are excluded

*AST* aspartate transaminase; *ALT* alanine transaminase

### Statistical Analyse

Statistical analyses were conducted using Stata/SE version 15.1 (StataCorp). The normality of the data was analyzed by histogram in terms of skewness and kurtosis. Continuous data are reported as mean ± standard deviation. Categorical variables are expressed as frequency and percentage. Statistical significance was calculated using analysis of variance for continuous data, and Fisher’s exact test was used for categorical variables. Multiple comparison procedure was used with Bonferroni method for continuous data and Hochberg method for categorical data. Logistic regression analysis is often used to calculate adjusted odds ratio, which approximates the adjusted relative risk when the outcome prevalence is low. However, odds ratio overestimates relative risk for more common outcomes (> 10 %) [7]. Therefore, to calculate the estimated relative risk (eRR) and 95 % confidence interval (CI), we used a Poisson regression analysis with a robust error variance. Maternal age and PC levels were included as covariates to assess the eRR for severe hypertension, utero-placental dysfunction, and maternal organ damage except thrombocytopenia. In addition to maternal age and PC levels, we included severe hypertension, utero-placental dysfunction, and maternal organ damage except for thrombocytopenia as covariates to assess the eRR for preterm delivery < 34 GW and NICU admission.

**Fig. 1:**
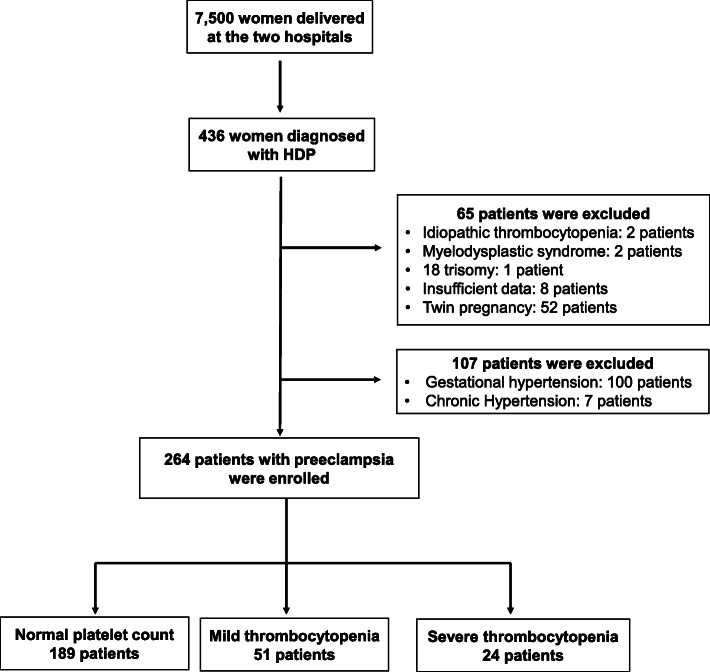
Schematic illustration of the patient selection criteria HDP: hypertensive disorders of pregnancy

## Results

There were 436 women who were diagnosed with HDP during the study period, and 172 of them fulfilled the exclusion criteria and were not included in the analysis. Therefore, 264 women were evaluated in this study (Fig. 1). PC levels were normal in 189 patients, 51 had mild thrombocytopenia, and 24 had severe thrombocytopenia. Table 2 shows the clinical characteristics based on PC levels. The clinical characteristics were not different between the three groups. Table 3 presents the perinatal outcomes and clinical features of pre-eclampsia based on PC levels. GW at the onset of pre-eclampsia (severe thrombocytopenia: 29.7 ± 5.5, normal: 34.1 ± 4.4, *p* < 0.001, mild thrombocytopenia: 33.7 ± 3.8, *p* = 0.001) and the day of delivery (severe thrombocytopenia: 31.4 ± 5.2, normal: 35.1 ± 3.9, *p* < 0.001, mild thrombocytopenia: 34.9 ± 3.6, *p* = 0.001) were significantly earlier in patients with severe thrombocytopenia although the difference was not detected between the patients with normal PC and those with mild thrombocytopenia. The prevalence of preterm delivery < 34 GW (severe thrombocytopenia: 66.7 %, normal: 34.9 %, *p* = 0.011, mild thrombocytopenia: 35.3 %, *p* = 0.028), NICU admission (severe thrombocytopenia: 87.5 %, normal: 53.4 %, *p* = 0.005, mild thrombocytopenia: 56.9 %, *p* = 0.019), and maternal organ damage except for thrombocytopenia (severe thrombocytopenia: 54.2 %, normal: 12.2 %, *p* < 0.001, mild thrombocytopenia: 11.8 %, *p* < 0.001) were significantly higher in patients with severe thrombocytopenia although the difference was not detected between the patients with normal PC and those with mild thrombocytopenia. The prevalence of severe hypertension and utero-placental dysfunction were equivalent between the three groups.

**Table 2:** Clinical characteristics based on the platelet count levels

	Normal (*n* = 189)	Mild (*n* = 51)	Severe (*n* = 24)	*P1*	*P2*	*P3*
Maternal Age (y.o)^a^	33.9 ± 5.2	34.5 ± 5.1	35.7 ± 4.8	1.000	0.333	1.000
Primipara^b^	115 (61.2)	33 (64.7)	15 (62.5)	1.000	1.000	1.000
Previous history of pre-eclampsia^b^	19 (10.1)	3 (5.9)	1 (4.2)	1.000	1.000	1.000
Low-dose aspirin for prevention^b^	2 (1.1)	2 (3.9)	1 (4.2)	0.597	0.605	1.000
Cesarean delivery^b^	146 (77.3)	41 (82.0)	22 (91.7)	0.565	0.355	0.565

^a^: mean ± standard deviation, statistical significance was calculated with analysis of variance and multiple comparison was conducted with Bonferroni method

^b^: n (%), statistical significance was calculated with Fisher’s exact test and multiple comparison was conducted with Hochberg method

Normal, platelet count ≥ 150 × 10^9^/L; Mild, platelet count < 150 × 10^9^/L and ≥ 100 × 10^9^/L; Severe, < 100 × 10^9^/L

*P1* normal versus mild; *P2* normal versus severe, *P3* mild versus severe

**Table 3:** Pre-eclampsia outcomes and clinical features based on the platelet count levels

	Normal (*n* = 189)	Mild (*n* = 51)	Severe (*n* = 24)	*P1*	*P2*	*P3*
GW at the onset of pre-eclampsia (weeks) ^a^	34.1 ± 4.4	33.7 ± 3.8	29.7 ± 5.5	1.000	< 0.001	0.001
GW at the day of delivery (weeks) ^a^	35.1 ± 3.9	34.9 ± 3.6	31.4 ± 5.2	1.000	< 0.001	0.001
Preterm delivery < 34 GW^b^	66 (34.9)	18 (35.3)	16 (66.7)	1.000	0.011	0.028
NICU admission^b^	101 (53.4)	29 (56.9)	21 (87.5)	0.752	0.005	0.019
Severe hypertension^b^	126 (66.7)	36 (70.6)	13 (54.2)	0.736	0.516	0.516
Utero-placental dysfunction^b^	54 (28.6)	17 (33.3)	7 (29.2)	1.000	1.000	1.000
Maternal organ damage except thrombocytopenia^b^	23 (12.2)	6 (11.8)	13 (54.2)	1.000	< 0.001	< 0.001

^a^: mean ± standard deviation, statistical significance was calculated with analysis of variance and multiple comparison was conducted with Bonferroni method

^b^: n (%), statistical significance was calculated with Fisher’s exact test and multiple comparison was conducted with Hochberg method

Normal, platelet count ≥ 150 × 10^9^/L; Mild, platelet count < 150 × 10^9^/L and ≥ 100 × 10^9^/L; Severe, < 100 × 10^9^/L

*P1*, normal versus mild; *P2*, normal versus severe, *P3*, mild versus severe

*GW* gestational weeks; *NICU* neonatal intensive care unit

Table 4 shows the age-adjusted eRRs for severe hypertension, utero-placental dysfunction, and maternal organ damage except for thrombocytopenia. Mild and severe thrombocytopenia were not related to severe hypertension and utero-placental dysfunction. Although mild thrombocytopenia was not associated with maternal organ damage except for thrombocytopenia (eRR: 0.97, 95 % CI: 0.41–2.26), severe thrombocytopenia increased the risk of maternal organ damage except for thrombocytopenia (eRR: 4.46, 95 % CI: 2.59–7.68). Table 5 represents the adjusted eRRs for preterm delivery < 34 GW and NICU admission. While mild thrombocytopenia was not related to preterm delivery (eRR: 0.91, 95 % CI: 0.62–1.35) and NICU admission (eRR: 0.97, 95 % CI: 0.76–1.24), severe thrombocytopenia increased the risk of preterm delivery < 34 GW (eRR: 1.61, 95 % CI: 1.06–2.45) and NICU admission (eRR: 1.35, 95 % CI: 1.06–1.73).

**Table 4:** Estimated relative risk of mild and severe thrombocytopenia for severe hypertension, utero-placental dysfunction, and maternal organ damage except thrombocytopenia

	eRR	95 % CI	*p* value
**Severe hypertension**			
Age^a^	1.02	0.93–1.11	0.689
Mild thrombocytopenia^b^	1.06	0.86–1.30	0.597
Severe thrombocytopenia^b^	0.81	0.55–1.18	0.273
**Utero-placental dysfunction**			
Age^a^	1.03	0.86–1.23	0.734
Mild thrombocytopenia^b^	1.16	0.74–1.82	0.512
Severe thrombocytopenia^b^	1.01	0.52–1.96	0.977
**Maternal organ damage except thrombocytopenia**			
Age^a^	0.99	0.79–1.25	0.949
Mild thrombocytopenia^b^	0.97	0.41–2.26	0.939
Severe thrombocytopenia^b^	4.46	2.59–7.68	< 0.001

^a^: Estimated relative risk of 5 years increase in age

^b^: Reference is normal platelet count (> 100 × 10^9^/L)

Mild thrombocytopenia, platelet count < 150 × 10^9^/L and ≥ 100 × 10^9^/L; severe thrombocytopenia, platelet count < 100 × 10^9^/L

*eRR* estimated relative risk; *CI* confidence interval

**Table 5:** Estimated relative risk of mild and severe thrombocytopenia for preterm delivery and NICU admission

	eRR	95 % CI	*p* value
**Preterm delivery < 34 GW**			
Age^a^	1.07	0.92–1.25	0.359
Mild thrombocytopenia^b^	0.91	0.62–1.35	0.649
Severe thrombocytopenia^b^	1.61	1.06–2.45	0.026
Severe hypertension	1.30	0.93–1.82	0.121
utero-placental dysfunction	2.22	1.66–2.96	< 0.001
maternal organ damage except thrombocytopenia	1.49	1.05–2.12	0.025
**NICU admission**			
Age^a^	1.09	0.99–1.20	0.071
Mild thrombocytopenia^b^	0.97	0.76–1.24	0.814
Severe thrombocytopenia^b^	1.35	1.06–1.73	0.016
Severe hypertension	1.17	0.94–1.44	0.158
utero-placental dysfunction	2.23	1.87–2.67	< 0.001
maternal organ damage except thrombocytopenia	1.50	1.22–1.84	< 0.001

^a^: Estimated relative risk of 5 years increase in age

^b^: Reference is normal platelet count (> 100 × 10^9^/L)

Mild thrombocytopenia, platelet count < 150 × 10^9^/L and ≥ 100 × 10^9^/L; severe thrombocytopenia, platelet count < 100 × 10^9^/L

*eRR* estimated relative risk; *CI* confidence interval; *GW* gestational weeks; *NICU* neonatal intensive care unit

## Discussion

The results of the present study revealed that mild thrombocytopenia (≥ 100 × 10^9^/L and < 150 × 10^9^/L) is not a risk factor for developing severe features of pre-eclampsia, including severe hypertension, maternal organ damage, and utero-placental dysfunction. Although physicians decide the timing of delivery in patients with pre-eclampsia based on GW and various clinical aspects in addition to PC counts, the rates of preterm delivery and NICU admission may be affected by the levels of PC counts, because thrombocytopenia is included as a sign of maternal organ damage. However, preterm delivery and NICU admission rates in patients with mild thrombocytopenia were not different from the rates in patients with normal PC. On the other hand, severe thrombocytopenia was a risk factor of developing maternal organ damage, except for thrombocytopenia. Although the rates of preterm delivery and NICU admission were higher in patients with severe thrombocytopenia than in patients with normal PC, the results might have been biased because severe thrombocytopenia is an indicator of pregnancy termination [1]. However, the onset of pre-eclampsia, which is not biased by PC levels, was earlier in patients with severe thrombocytopenia than in patients with normal PC. Early onset of pre-eclampsia is associated with worse outcomes in both mother and baby [8]. These findings support that PC levels ≥ 100 × 10^9^/L and < 150 × 10^9^/L does not reflect a sign of maternal organ damage in pre-eclampsia.

Gestational thrombocytopenia (GT) is defined as PC levels < 150 × 10^9^/L, with the exclusion of other possible diagnoses [5]. GT occurs in 4.4–11.6 % of pregnancies and accounts for almost 75 % of thrombocytopenia in pregnancy [9–11]. Since there are no available biomarkers to provide a definite diagnosis of GT, some cases of thrombocytopenia detected in patients with HDP may not reflect the disease progression and may be a result of GT. In addition, only 1–5 % women with GT develop PC levels < 100 × 10^9^/L, and GT does not usually affect perinatal outcomes [5]. Therefore, this could explain why mild thrombocytopenia was not associated with worse perinatal outcomes in our study, as thrombocytopenia was not caused by pre-eclampsia.

Blood coagulation disorders, including thrombocytopenia, are a sign of maternal organ damage; therefore, patients with thrombocytopenia are considered as severe pre-eclampsia even with blood pressure levels < 160/110 mmHg and no other signs of maternal organ damage [1, 2]. If the majority of cases of mild thrombocytopenia are not associated with worse maternal and neonatal outcomes, using PC levels < 150 × 10^9^/L as a cut-off value of a sign of maternal organ damage may increase unnecessary hospitalization and iatrogenic preterm delivery, particularly after 34 GW.

Pre-eclampsia is a progressive disease and PC levels may decrease as the disease progresses. Our findings indicate that mild thrombocytopenia is not a severe feature of pre-eclampsia; however, PC levels may decrease in patients with mild thrombocytopenia as pre-eclampsia progresses and become severe thrombocytopenia. Evaluation of the speed of PC decrease and the rate of PC decrease from the baseline will give further information to predict the deterioration of pre-eclampsia. Previous studies have reported that platelet indices, such as mean platelet volume and platelet distribution width, were associated with pre-eclampsia development and severity [12–14]. Analysis of platelet indices in addition to PC may help identify patients with mild thrombocytopenia in which PC levels will progressively decrease.

In Japan, thrombocytopenia was not included in the criteria of diagnosis and severity of pre-eclampsia until May 2018. The protocol for HDP patient management in Hokkaido University Hospital and Japan Community Health Care Organization Hokkaido Hospital was not changed after the Japanese criteria revision. Therefore, clinicians’ decisions would not have been affected the discrepancy in PC cut-off value in the present study.

This study has several limitations. This was a retrospective study; therefore, the timing of termination was decided based on the clinical decisions of the doctors in each hospital. Although serum biomarkers, such as the ratio of soluble fms-like tyrosine kinase to placental growth factor, are associated with development of pre-eclampsia [15], they were not widely available in Japan during the study period. Thus, we were unable to examine the association between mild thrombocytopenia and those biomarkers. Finally, low-dose aspirin is useful for prevention of pre-eclampsia in high-risk patients [16]. However, the rate of low-dose aspirin usage was very low in our study. Therefore, it is difficult to determine whether our findings are adaptable for patients using low-dose aspirin.

## Conclusion

.

Despite these limitations, this study implies that mild thrombocytopenia in pre-eclampsia is not related with severe features of pre-eclampsia, and would not be suitable as a sign of maternal organ damage in patients with pre-eclampsia. PC levels < 100 × 10^9^/L may be better cut-off value as a sign of maternal organ damage in pre-eclampsia.

## Data Availability

The datasets used and/or analysed during the current study will be available from the corresponding author on reasonable request.

## Abbreviations

CI: Confidence interval
GT: Gestational thrombocytopenia
GW: Gestational weeks
HDP: Hypertensive disorders of pregnancy
NICU: Neonatal intensive care unit
eRR: Estimated relative risk
PC: Platelet count
